# Using fluorescent *in vitro* amphibian cell infection models to quantify pathogenicity of *Batrachochytrium dendrobatidis*

**DOI:** 10.1016/j.ymeth.2026.02.006

**Published:** 2026-02-13

**Authors:** Kashmini K Sumanasekera, Lee Berger, Andrea L Vu, Jacques Robert, Nazia Akram, Lee F Skerratt, Francisco De Jesús Andino, Rebecca J Webb

**Affiliations:** a Melbourne Veterinary School, University of Melbourne, Melbourne, Victoria, Australia; b Department of Microbiology and Immunology. University of Rochester Medical Center, Rochester, NY, United States

**Keywords:** *In vitro* model, *Batrachochytrium dendrobatidis*, A6, Host, Disease, Cell

## Abstract

Chytridiomycosis is a devastating amphibian fungal disease and methods to understand host pathogen interactions and test novel mitigation strategies are urgently needed. A recently developed *in vitro* frog cell infection model offers an efficient and ethical approach for chytridiomycosis research, allowing precise manipulation of host and fungal traits. However, reliable assays are needed to quantify disease outcomes. Here, we validate methods to quantify chytridiomycosis severity in an *in vitro* model by measuring pathogen loads and cytopathic effects on host cells. We assessed the ability of methods to distinguish fungal loads by comparing results of exposure to various *Batrachochytrium dendrobatidis* (*Bd*) infectious doses incubated under optimal and suboptimal conditions for fungal growth. Using a genetically modified strain of fluorescent *Bd* allowed quantification of fungal burden via microscopy, spectrophotometry and flow cytometry. For host cell effects, a DAPI staining protocol quantified cell damage and a modified MTT assay quantified cell viability. Our work provides a toolbox of methods to utilise *in vitro* cell infection models to investigate the function and effect size of fungal virulence and host resistance factors, enabling diverse research aimed at understanding and mitigating chytridiomycosis.

## 1. Introduction

*In vitro* infection models are increasingly used as alternatives to *in vivo* animal experiments, as they address ethical concerns and regulatory frameworks such as the 3Rs (Replacement, Reduction, and Refinement). *In vitro* models are cost-effective, efficient, reproducible and avoid use of sentient animals, allowing for more replicates and easy manipulation of host and pathogen factors, as well as manipulation of environmental variables that would be difficult to achieve with *in vivo* models [1]. Due to their simplicity, the contribution of individual traits can be assessed, making them ideal for pathogenesis research such as characterising fungal virulence factors [2].

Chytridiomycosis is a deadly amphibian disease caused by the fungus *Batrachochytrium dendrobatidis* (*Bd*) that has caused catastrophic declines and extinctions of amphibians worldwide [3]. *Bd* has two life stages, the free-living flagellated motile zoospore stage which infects amphibian epidermis, where it develops into the second stationary, intracellular zoosporangium stage. Once mature, the zoosporangia asexually reproduce and release zoospores via discharge tubes back to the environment or to infect the same host [4]. *Bd* infection disrupts cutaneous osmoregulatory mechanisms causing electrolyte loss leading to the death of susceptible amphibian hosts [5]. Chytridiomycosis has been recorded in approximately 700 amphibian species in a diversity of habitats globally [6,7]. Temperature, salinity, pH, and moisture are important conditions for the survival, growth, and virulence of the fungus [8,9]. *Bd* isolates can differ in their thermal performance [10,11], but generally display optimal growth between 17 and 25 °C, failing to grow above 28°C [8,9].

Despite the global efforts, there is no broadly applicable solution to reduce chytridiomycosis in wild amphibians. Several novel promising approaches are under development, including environmental manipulation and synthetic biology to enhance host disease resistance, and pathogen focused methods to reduce fungal virulence [12–14]. Research on host-pathogen interactions and disease mitigation strategies have been conducted *in vitro* using standard tryptone cell culture media and *in vivo* in live animals. However, culturing *Bd* in tryptone does not replicate the host environment [4,15,16], while *in vivo* studies raise significant ethical concerns and are limited by challenges in manipulation of variables. Moreover, live animal experiments are costly, space-intensive, and time-consuming, which limit their replication for verification. Therefore, developing more realistic, modifiable, and animal welfare-friendly *in-vitro* amphibian cell infection models could accelerate and streamline chytridiomycosis research.

Recently, amphibian cell line infection models have been developed utilizing *Bd* [17] as well as its sister species, the salamander infecting *Batrachochytrium salamandrivorans* (*Bsal*) [18], in *Xenopus laevis* kidney epithelial cells (A6 cell line) and lung fibroblasts (DWJ cell line) [19]. The complete life cycle of both *Bd* and *Bsal* have been demonstrated in A6 cells, including host cell penetration, growth, maturation, host cell death, and fungal release. Real time observations and imaging of *Bd* infection and growth have been successfully demonstrated using transgenic *Bd* constitutively expressing red fluorescent protein [19]. These models could be utilised to define host-pathogen interactions and test the outcomes of novel treatment strategies for chytridiomycosis. However, to achieve this goal, reliable methods to quantify chytrid growth and pathogenesis are needed. Current methods for measuring *Bd* growth [20] and viability in tryptone based media − such as optical density [21], methylene blue colorimetry [22], trypan blue [23], and MTT assays [24] − cannot be applied in this infection model due to interference from A6 cells, and using qPCR is complicated due to DNA carryover from the zoospore inoculum [18]. Likewise, traditional methods to quantify A6 cell viability (such as MTT) are complicated by the presence of *Bd*, which also reacts to these compounds. Therefore, we sought to develop and evaluate a range of alternative specific methods to quantify different aspects of *Bd* growth, infection and pathogenesis using a fluorescent *in vitro* amphibian cell infection model.

## 2. Methods

### 2.1. A6 cell culture

The immortalised A6 epithelial cell line (ATCC CCL-102) generated from an outbred *Xenopus laevis* kidney was employed for this model. Cells were cultured in “A6 media”, containing 75% NCTC 109 media (Gibco, Thermo Fisher Scientific, Waltham, MA, USA) supplemented with 25% Fetal Bovine Serum (FBS) (Gibco, Thermo Fisher Scientific, Waltham, MA, USA) and 2 mM glutamine (Gibco, Thermo Fisher Scientific, Waltham, MA, USA) in 25 cm^2^ flasks at 27 °C with 5% CO_2_. A 5 min incubation at 27 °C with 0.25% trypsin-EDTA (Gibco, Thermo Fisher Scientific, Waltham, MA, USA) was used to passage and transfer cells to plates for experiments.

### 2.2. Bd transformation, inoculation and experimental design

To enable fluorescent quantification, *Bd* was transformed to express red fluorescent protein and maintained in TGhL media containing 5 μg/ml hygromycin as previously described [19]. Pure zoospore suspensions were collected from mature cultures by incubating the zoosporangia monolayer with fresh TGhL for 1 h, centrifugation at 2500 × g, and resuspension of the zoospore pellet in solution C (20% Leibovitz’s L-15 Medium (Gibco, Thermo Fisher Scientific, Waltham, MA, USA), 77.5% water, 2.5% FBS) [19]. For all experiments, a spectrum of *Bd* growth was achieved by inoculating A6 cells with three multiplicities of infection (MOI) concentrations of *Bd* [19], as well as an uninfected control, and incubating at either 19 °C (supports *Bd* growth) or 27 °C (inhibits *Bd* growth). Cells were inoculated with *Bd* zoospores in solution C [19] for 1 hr, after which the inoculation media was removed, replaced with solution A (70% Leibovitz’s L-15 Medium, 20% water, 10% FBS) [17]. All experiments were repeated at least twice to confirm results, the data presented represents three technical replicate wells per condition from the final experiment.

### 2.3. Image J to quantify fluorescent Bd loads

Two clear 48 well plates were seeded with A6 cells at 50,000 cells per well and incubated at 27 °C in A6 media for 18 hr. Three replicate wells were inoculated with zoospores at 0, 1, 2, or 4 MOI for 1 hr at room temperature, after which the inoculation media was removed, replaced with 300 μL of solution A and incubated at 19 °C or 27 °C for 7 d. The surface area of *Bd* in the cells was measured by imaging under red fluorescence using an EVOS inverted microscope. Each well was imaged at three locations at 40x magnification with 50% intensity and 100% contrast without scale bars. This results in imaging across the majority of the well, thus, removes bias from image selection. Images were analysed using ImageJ web browser using default threshold (set to dark background) and the “measure particle” function to calculate the percent area of fluorescent signal. Fluorescent signal of the infected wells was calculated relative to the uninfected control average for each temperature.

### 2.4. Fluorescent spectrophotometry to quantify Bd loads

Two black 96 well plates were seeded with A6 cells at 25,000 cells per well and incubated at 27 °C in A6 media for 18 hr. Three replicate wells were inoculated with zoospores at 0, 1, 2, and 4 MOI for 1 hr at room temperature, after which the inoculation media was removed, replaced with 100 μL of solution A, and incubated at 19 °C or 27 °C for 4 d. The fluorescence intensity was measured using a Thermo Scientific^™^ Varioskan^™^ LUX modular multi-technology microplate reader at 554 nm excitation and 581 nm emission, calculated relative to the uninfected control.

### 2.5. Flow cytometry to quantify the frequency of infected A6 cells

Two clear 48 well plates were seeded with A6 cells at 50,000 cells per well and incubated at 27 °C in A6 media for 18 hr. Three replicate wells were inoculated with zoospores at 0,1,2, or 3 MOI at either 19 °C or 27 °C for 1 hr, after which the inoculation media was removed, replaced with 300 μL of solution A, and incubated at 19 °C or 27 °C. After 24 hr, excess media was removed, and the A6 cells were washed with 200 μL APBS, detached with 0.25% trypsin-EDTA, centrifuged and resuspended in 300 μL FACS staining buffer (APBS containing 1% BSA and 0.05% sodium azide). Each sample was mixed before running 10,000 events on a BD Accuri C6 Plus Flow Cytometry. Using uninfected cells to gate side scatter (SSC-A) vs forward scatter (FSC-A), following single cells gating to select single cells only and exclude doublets and the proportion of *Bd*-infected A6 cells was determined using PE channel to detect A6 cells with red fluorescence.

### 2.6. DAPI to quantify A6 cell health

Two clear 96 well plates were seeded with A6 cells at 25,000 cells per well and incubated at 27 °C in A6 media for 18 hr. Three replicate wells were inoculated with zoospores at 0, 0.5, 1 and 2 MOI at either 19 °C or 27 °C for 1 hr, after which the inoculation media was removed, replaced with 100 μL solution A, and incubated at 19 °C or 27 °C for 3 d. A6 cells were stained by the gentle addition of 50 μL sterile filtered 0.5 μg/mL DAPI (4′,6-diamidino-2-phenylindole) (Invitrogen) for 5 min (final concentration 0.16 μg/mL), after which the staining solution was gently removed and replaced with 100 μL of solution A. Cell damage was measured immediately by imaging under blue fluorescence using an EVOS microscope. Each well was imaged at two locations at 40x magnification with 100% contrast without scale bars. Images were analysed using the ImageJ web browser using manual threshold (set to dark background) and the “measure particle” function to calculate the total number of fluorescent nuclei. The number of DAPI positive nuclei from infected cells were calculated relative to the uninfected control wells. To understand the progression of cell damage during infection, an additional experiment was conducted in which uninfected and infected (2 MOI) cells were stained at multiple timepoints compared to a formalin fixed uninfected control well to calculate proportion of damaged cells. To determine whether dead *Bd* cells would interfere with this assay, we infected A6 cells at 1 MOI, incubated at 19 °C for 3 d and added 5 μg/ml Terbinafine hydrochloride (Lamisil) for 18 hr prior to staining with DAPI.

### 2.7. MTT to quantify A6 cell metabolic activity

Two clear 96 well plates were seeded with A6 cells at 25,000 cells per well and incubated at 27 °C in A6 media for 18 hr. Three replicate wells were inoculated with zoospores at 0, 1, 2, and 4 MOI for 1 hr at room temperature, after which the inoculation media was removed, replaced with 100 μL solution A, and incubated at 19 °C or 27 °C for 7 d. As MTT can react to *Bd* metabolism [24], both plates were treated with 5 μg/ml Terbinafine hydrochloride and incubated at 30°C for 18 hr to inactivate the *Bd*. After incubation, the media was replaced with 100 μL of solution A. A6 cell activity was quantified using the CyQUANT^™^ MTT Cell Proliferation Assay (Thermo Fisher), prepared as per the manufacturer’s instructions and 10 μL was added to each well and incubated at 30 °C for 4 h. Excess media was carefully removed without disturbing the crystals at the bottom of the wells, and 50 μL DMSO was added to each well, and the eluted dye absorbance was measured using a Thermo ScientificTM VarioskanTM LUX modular multi-technology microplate reader at 540 nM, and calculated relative to the uninfected control. To ensure the *Bd* inactivation protocol was successful, we compared formazan production in infected cells with and without antifungal treatment.

### 2.8. Crystal violet staining to quantify A6 cell density

Two clear 48 well plates were seeded with A6 cells at 50,000 cells per well and incubated at 27°C in A6 media for 18 hr. Three replicate wells were inoculated with zoospores at 0, 0.5, 1, and 2 MOI for 1 hr at room temperature, after which the inoculation media was removed, replaced with 300 μL of solution A, and incubated at 19 °C or 27 °C for 8 d. A6 cells were fixed with formalin, washed, then stained with 0.1% crystal violet for 5 mins and washed again. A6 cell surface area was measured by imaging under trans illumination using a EVOS microscope. Each well was imaged at three locations at 40x magnification with 40% intensity and 50% contrast. Images were analysed using ImageJ web browser (set to light background) and the measure particle function to calculate the total area of stained cells relative to the uninfected control wells. Cell size was used as a measure of cell health as cell shrinkage is a typical sign of apoptosis [25]. After imaging, bound crystal violet was eluted with 400 μL methanol, transferred to a round bottom 96 well plate, and measured on a spectrophotometer at 570 nM.

### 2.9. Statistical analysis

All data was analysed using GraphPad prism software (10.2.3). Simple linear regressions were conducted to describe the association between zoospore inoculum (MOI) and *Bd* load / host cell health parameters. One-way ANOVA was applied only on graphs where visual patterns in the data required statistical confirmation of differences among points. When ANOVA identified significant effects (p < 0.05), Tukey’s HSD post-hoc test was used to resolve pairwise differences.

## 3. Results

### 3.1. Quantifying Bd loads in A6 cells via Bd fluorescent signal

**Fig. 1.**
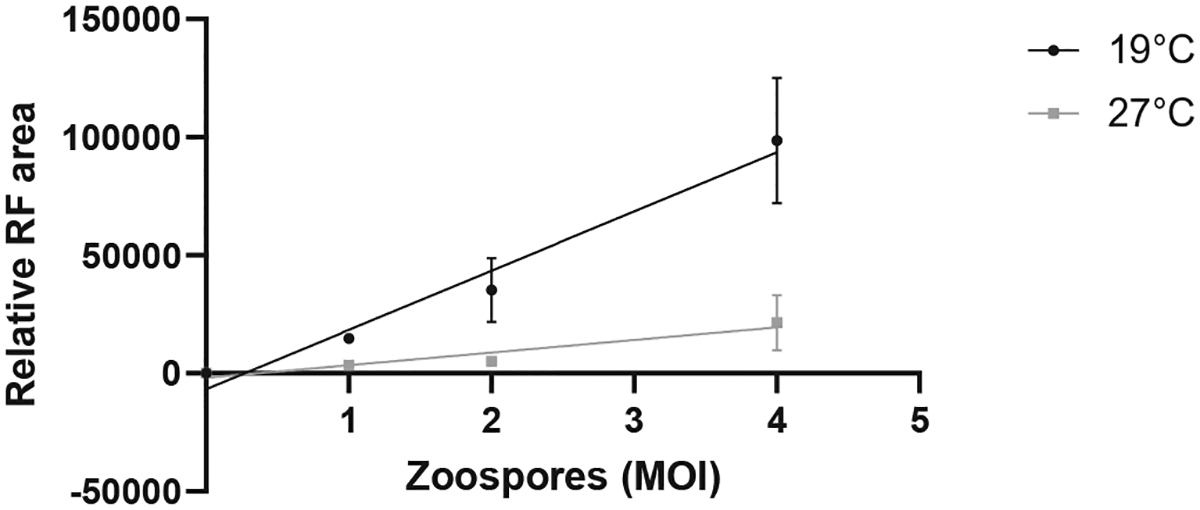
*Bd* loads estimated using Image J. *Xenopus laevis* kidney A6 cells were infected with red fluorescent (RF) *Bd* zoospores at 1, 2, and 4 MOI and incubated at 19°C or 27 °C. Each well was imaged under red fluorescence at day 7 and analysed using Image J to calculate total surface area of red fluorescence (RF). Linear regression of the relative RF area of three technical replicates is shown with standard deviations (19 °C R^2^ = 0.8812, 27 °C R^2^ = 0.6846).

**Fig. 2.**
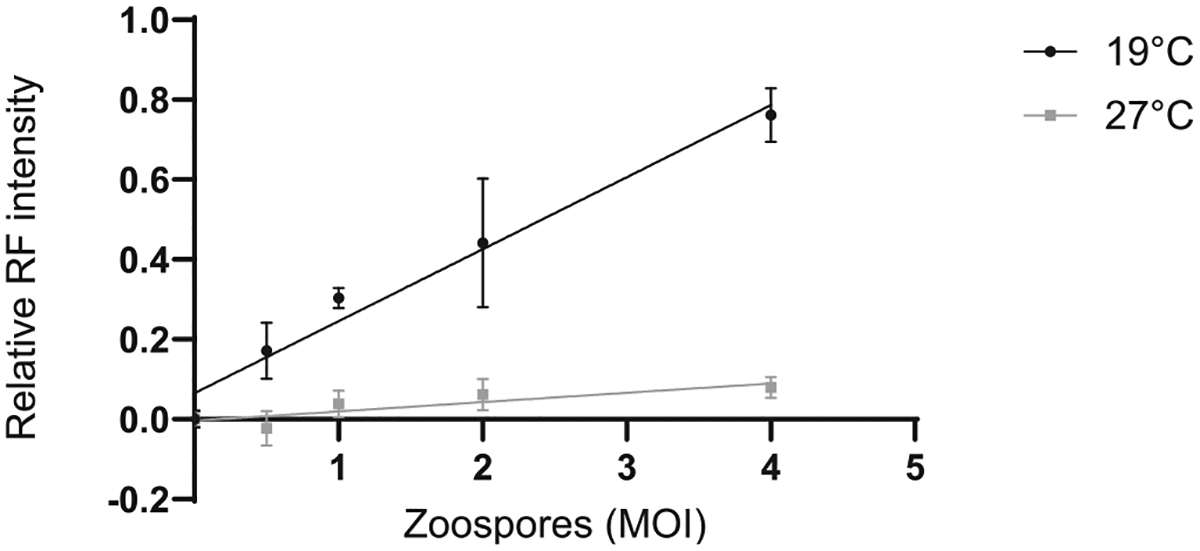
*Bd* loads estimated using fluorescence spectrophotometry. *Xenopus laevis* kidney A6 cells were infected with red fluorescent (RF) *Bd* zoospores at 1, 2, and 4 MOI and incubated at 19°C or 27°C. Fluorescence intensity of each well was measured using spectrophotometry at 554 nm excitation and 581 nm emission on day 4. Linear regression of the relative RF intensity of three technical replicates is shown with standard deviations (19°C R^2^ = 0.9076, 27°C R^2^ = 0.4980)

**Fig. 3.**
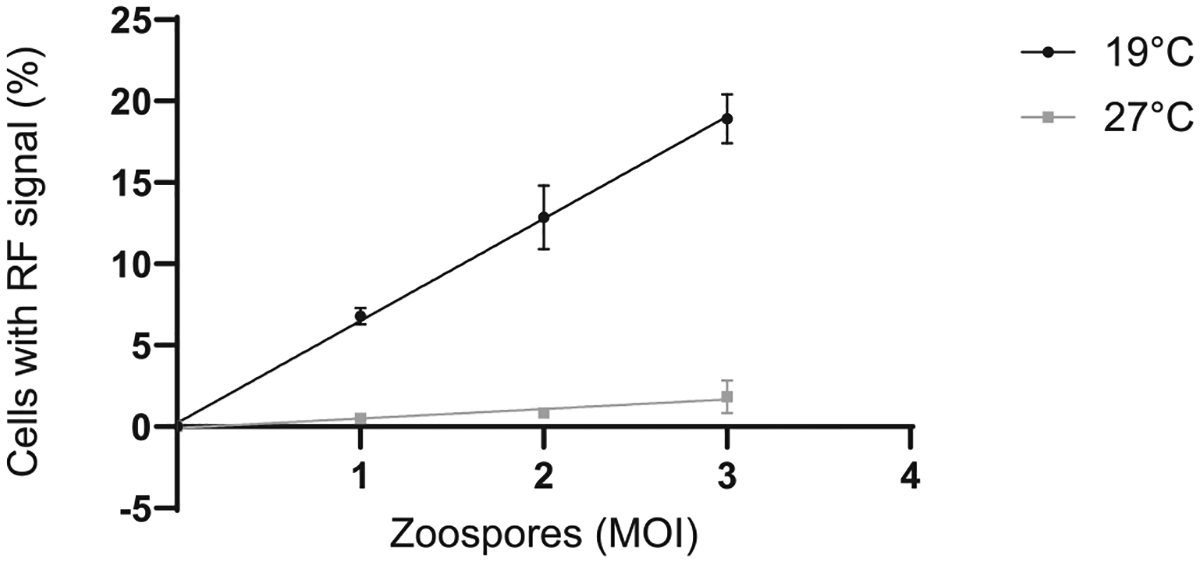
Frequency of A6 cells infected by *Bd* determined by flow cytometry. *Xenopus laevis* A6 cells were infected with red fluorescent (RF) *Bd* zoospores at 1, 2, and 3 MOI and incubated at 19 °C or 27 °C. Cells were analysed by flow cytometry at 24 hr. Gating was based on FSC-A vs SSC-A using uninfected controls, followed by single-cell gating. A6 cells infected with *Bd* were quantified using red fluorescence in the PE channel. Linear regression of the proportion of cells with RF signal of three technical replicates is shown with standard deviations (19 °C R^2^ = 0.9782, 27 °C R^2^ = 0.6804).

Capturing images using a fluorescent microscope allowed quantification of *Bd* surface area. The relative area of red fluorescent (RF) signal increased in proportion to the zoospore infective dose (Fig. 1). Similarly, measuring fluorescent signal using a spectrophotometer also detected differences in *Bd* loads (Fig. 2). As these methods are non-destructive, fluorescence can be measured at multiple time points on the same cells. In our experiments we chose 7 days and 4 days post-*Bd* infection to measure using imaging and spectrophotometry, respectively. The optimal time point for these measurements should be adapted for specific experiments depending on the rate of *Bd* growth due to the conditions or *Bd* isolate used. Flow cytometry was also effective for determining the frequency of infected A6 cells, as the percentage of A6 cells with a red fluorescent signal increased in proportion to the zoospore infective dose (Fig. 3). When A6 cells were incubated at a temperature beyond *Bd* thermal optimum but within the physiological range for A6 cells (27 °C), the fluorescent signal remained low for all three methods, consistent with no *Bd* growth (Figs. 1–3). However, due to the long half-life of RF in the zoospore inoculum, there was some background signal present even if the zoospores did not grow.

### 3.2. Quantifying damage, surface area and activity of A6 cells during infection

**Fig. 4.**
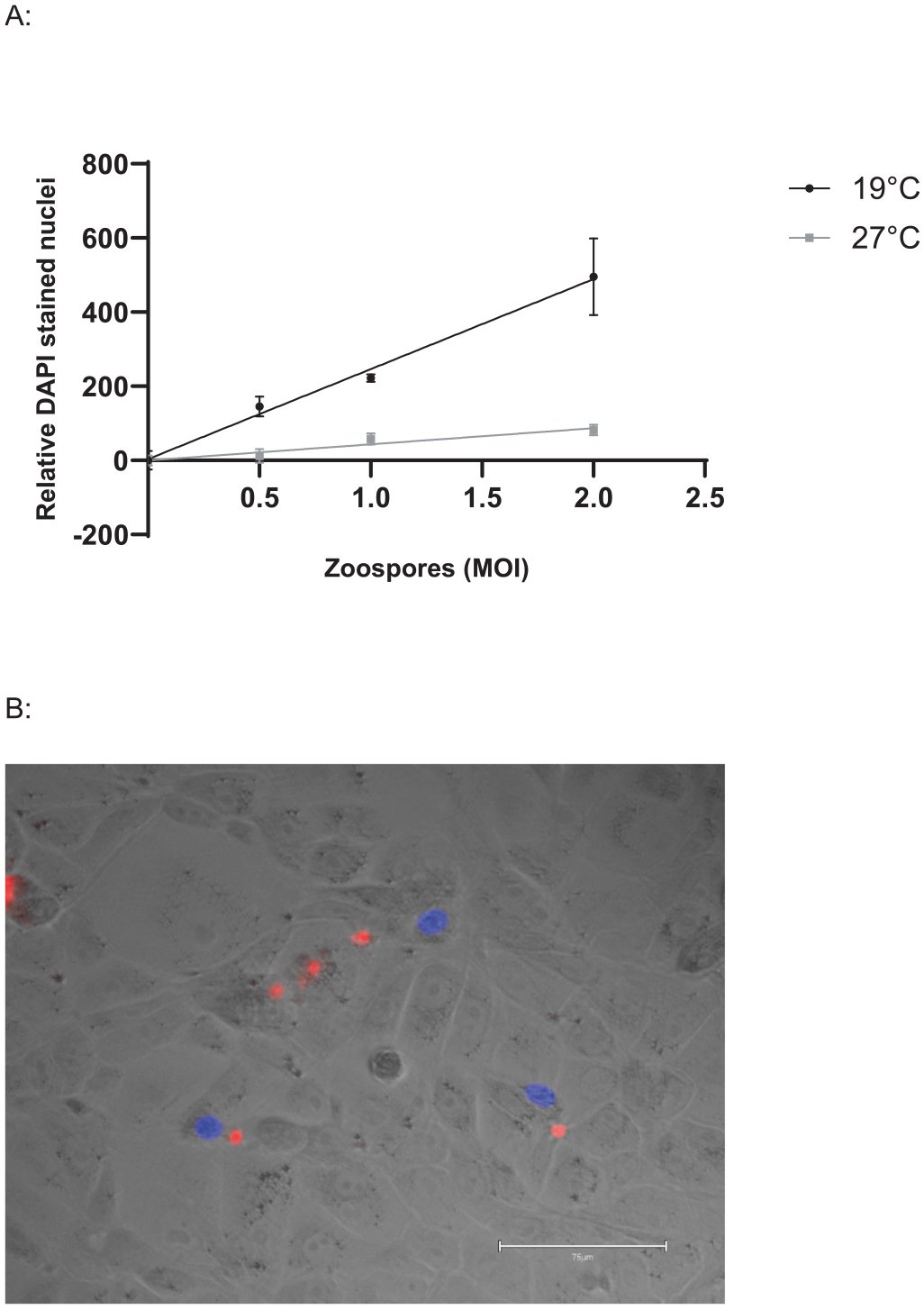
*Bd*-induced cell damage estimated by DAPI staining. A: Xenopus laevis A6 cells were infected with red fluorescent (RF) Bd zoospores at 0.5, 1, and 2 MOI and incubated at 19°C or 27 °C for 3 d. Damaged cells were stained with 0.16 μg/mL DAPI (4′,6-diamidino-2-phenylindole) for 5 mins before imaging and analysis with Image J. Linear regression of the relative number of DAPI stained nuclei of 3 technical replicates is shown with standard deviations (19 °C R^2^ = 0.9338, 27 °C R^2^ = 0.8105). B: Representative image of A6 cells exhibiting positive DAPI staining (blue) associated with zoosporangia (red), the scale bar is 75 μm.

**Fig. 5.**
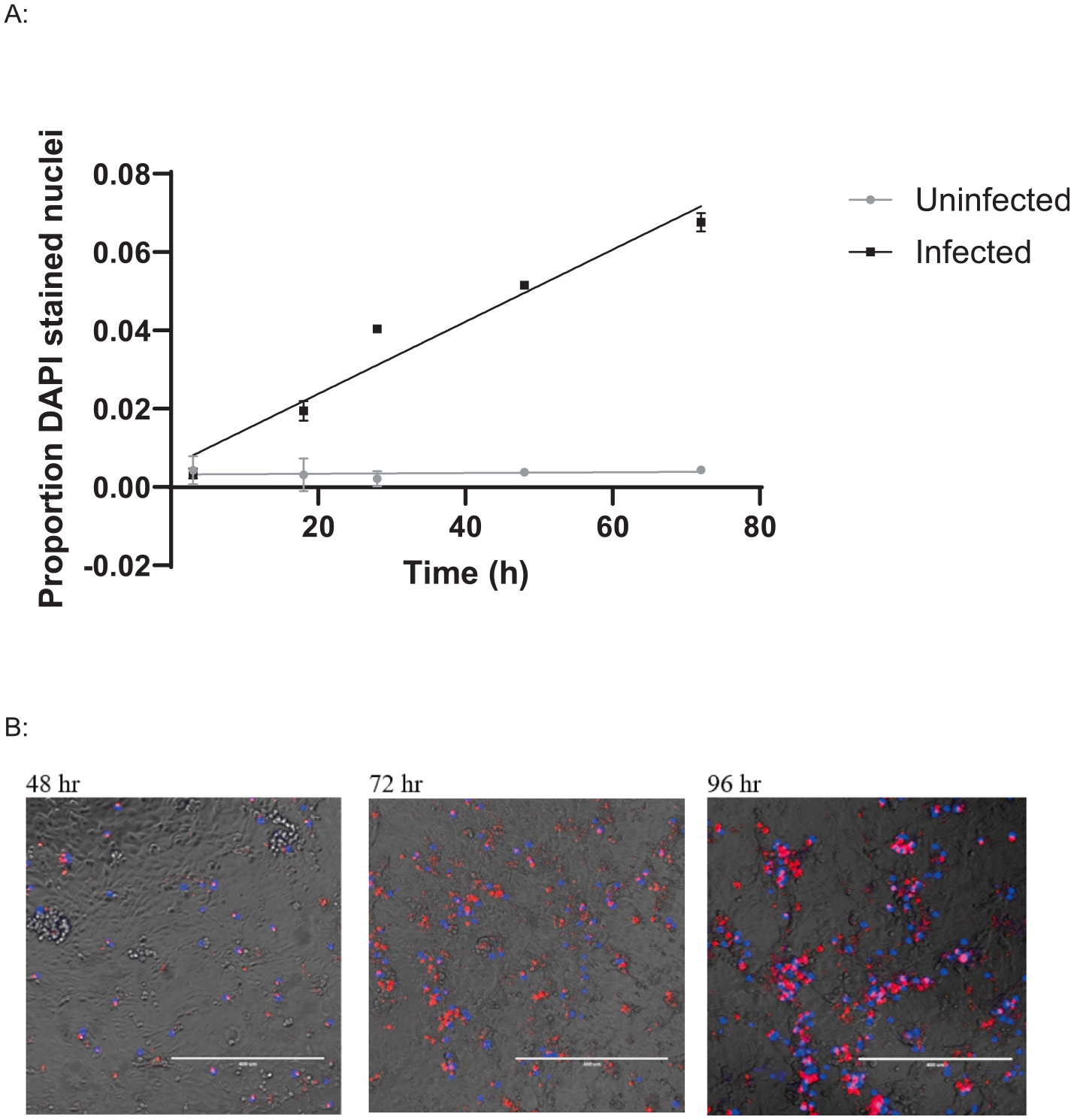
Use of DAPI staining to monitor disease progression. A: *Xenopus laevis* A6 cells were infected with red fluorescent (RF) *Bd* zoospores at 2 MOI and stained with 0.16 μg/mL DAPI at various time points after zoospore exposure to quantify cell damage compared to a formalin fixed uninfected control well. Linear regression of two technical replicates per condition per time point is shown with standard deviations (Uninfected R^2^ = 0.012, Infected R^2^ = 0.9458). B: Representative images taken at 48 hr, 72 hr and 96 hr after infection, the scale bar is 400 μm.

DAPI is a widely used DNA specific fluorescent dye, which can function as a live/dead stain when used at low concentrations due to the impermeability of healthy cell membranes [26]. Brief application of dilute DAPI appears to act as a marker of *Bd* induced cell damage. A6 cells with DAPI stained nuclei were frequently observed adjacent to *Bd* zoosporangia (Fig. 4b), and the number of DAPI stained host cell nuclei increased in proportion to the zoospore dose when cells were incubated at 19 °C. In contrast, DAPI staining remained stable when cells were incubated at 27 °C (Fig. 4a), indicating that zoospore nuclei were not detected using this method. DAPI positive cells were first detected at 18 hr post exposure and increased over the 96 hr experiment (Fig. 5). DAPI will also stain dead zoosporangia, both in TGhL (Supplementary Fig. 1) and within A6 cells (Supplementary Fig. 2).

**Fig. 6.**
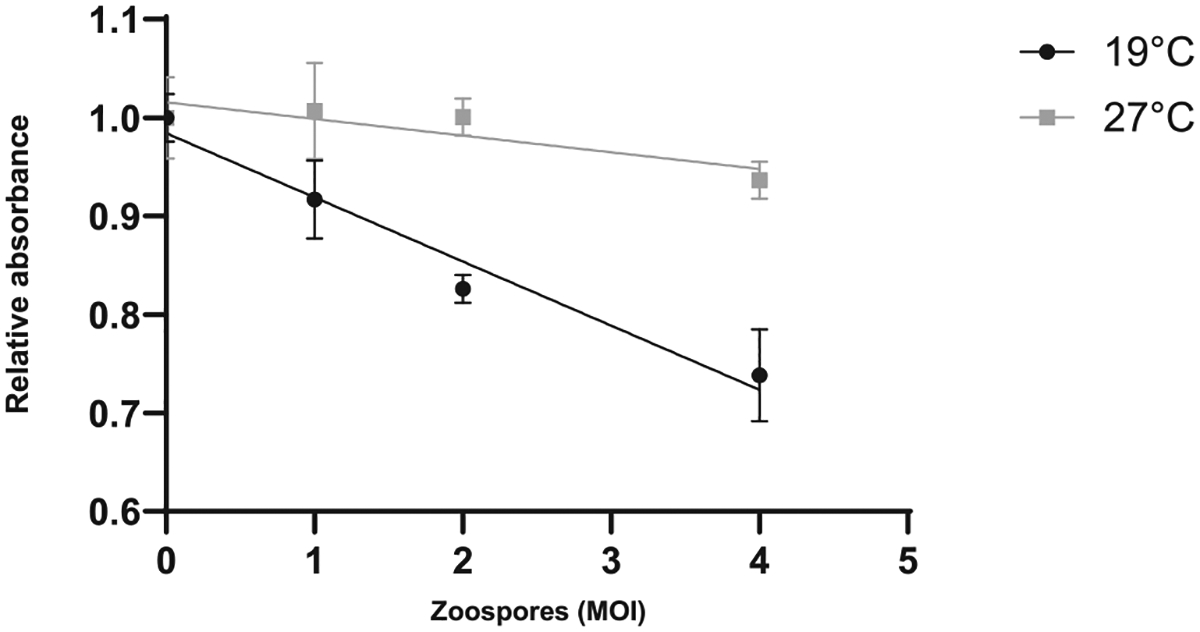
Pathogenic effects of *Bd* infection on A6 cells estimated by MTT cell viability assay. *Xenopus laevis* A6 cells were infected with *Bd* zoospores at 1, 2, and 4 MOI, and incubated at 19°C or 27 °C. On Day 7, both plates were treated with 5 μg/mL Terbinafine followed by 18 hr incubation at 30 °C to inactivate *Bd*, and A6 cell metabolic activity estimated using a commercial MTT assay (CyQUANT^™^). Linear regression of the relative absorbance of three technical replicates is shown with standard deviations (19 °C R^2^ = 0.8969, 27 °C R^2^ = 0.3849).

MTT (3-(4,5-dimethylthiazol-2-yl)-2,5-diphenyltetrazolium bromide) tetrazolium reduction assay is a commonly used cell viability assay, where viable cells with active metabolism convert MTT into a purple coloured formazan product [27]. Using this assay, relative A6 cell viability decreased with increasing *Bd* infective doses in cells incubated at 19 °C (Fig. 6). At 27 °C, host cell health showed a slight decline with increasing zoospore concentration, however, one-way ANOVA found no significant differences among concentrations (F = 2.76, df = 3, 8, p = 0.112). We used an antifungal treatment consisting of terbinafine and heat applied prior to the assay to neutralise *Bd* metabolic activity. Without antifungal treatment, *Bd* cells accumulated formazan crystals, reducing the sensitivity of the assay (Supplementary Fig. 3).

**Fig. 7.**
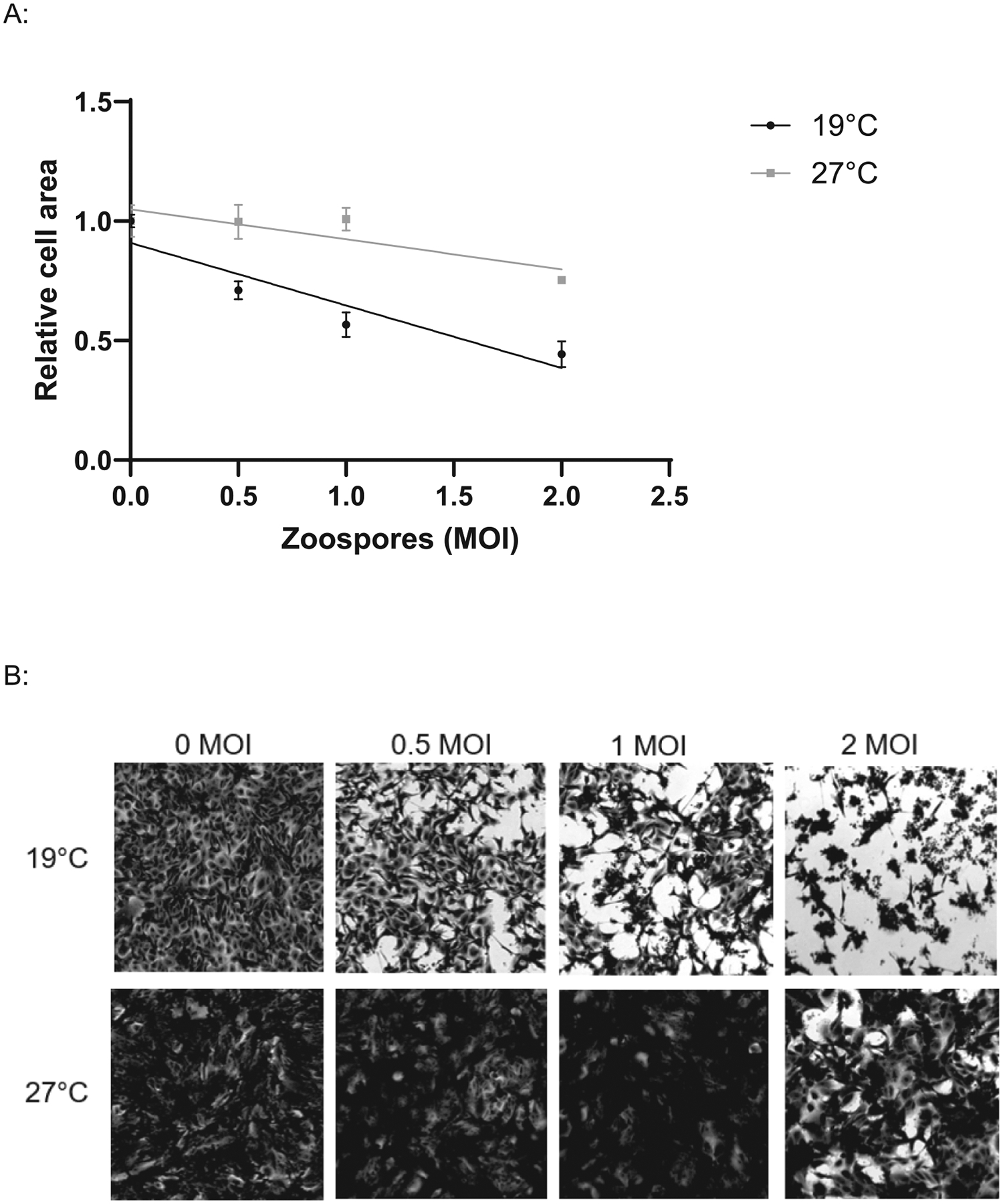
Use of crystal violet to detect differences in A6 cell surface area in *Bd* infection. A: *Xenopus laevis* A6 cells were infected with *Bd* zoospores at 0.5, 1 and 2 MOI and incubated at 19°C or 27 °C. On day 8 wells were fixed and stained with crystal violet and imaged to calculate relative A6 cell surface area using Image J calculated relative to the uninfected control. Linear regression of relative A6 surface area of three technical replicates is shown with standard deviations (19 °C R^2^ = 0.8436, 27°C R^2^ = 0.6358). B: Representative images of cells at 40x magnification.

Another commonly used assay is crystal violet, a stain that binds to DNA and proteins to determine the viability of cell monolayers [28]. When incubated at 19°C, following fixation and staining, we observed a decrease in A6 cell surface area correlating with increasing infective zoospore dose (Fig. 7). In contrast, when incubated at 27 °C, only a slight decrease in surface area was observed in A6 cells infected with the highest zoospore dose (Fig. 7). Although host cell area was significantly affected by zoospore concentration (ANOVA: F = 15.24, df = 3, 8, p = 0.0011), Tukey’s HSD post-hoc comparisons showed that only the highest zoospore concentration (2 MOI) significantly reduced cell area compared to 0, 0.5, and 1, while lower concentrations did not differ from 0. Overall, at 27 °C, cell area remained stable when exposed to low zoospore concentrations but declined at the highest concentration. Elution of crystal violet showed no difference between any of the treatments (data not shown). Fixed *Bd* cells stained very strongly with crystal violet, which likely contributed to the lack of sensitivity of this method.

## 4. Discussion

The recent development of *in vitro* cell infection models offers great benefits for chytridiomycosis research. Here, we extend their usefulness by validating various methods to easily quantify infection loads and disease severity (Table 1). These methods are key to assessing the function and effect size of host and pathogen factors.

Using fungi modified to constitutively express fluorescent protein has been used to monitor infection in plants [29], and here we show that it enables easy quantification of *Bd* burden using either image acquisition or fluorescent spectrophotometry, and the percentage of infected cells can be quantified using flow cytometry. Microscopy and spectrophotometry are also advantageous by enabling real-time and repeated measurements of *Bd* growth without nucleic acid extraction, PCR, sample fixation or staining. Further, in cases where transforming *Bd* to express fluorescent proteins is unsuitable, we anticipate that alternative fluorescent labels for *Bd*, such as cell-tracking dyes [18], or fixation and staining with calcofluor white [17] could be similarly quantified. Flow cytometry requires destructive sampling but offers the possibility of cell sorting to examine factors in infected vs uninfected cells or conduct transcriptomics.

In addition to quantifying pathogen burden, we also validated methods to detect host cell damage and viability, which enables quantification of “disease” in the model. A low concentration of DAPI briefly applied to unfixed cells appears sufficient to identify *Bd*-induced cell damage. In general, only the nuclei of A6 cells near invading *Bd* were stained (Fig. 4a), and the number of DAPI positive nuclei increased in proportion with increasing infectious dose (Fig. 4b). At low concentrations, DAPI cannot cross intact cell membranes, and we hypothesise that the A6 nuclei are stained as a result of damage caused by invading *Bd*. Using this protocol, we detected host cell damage from 18 hr after exposure to *Bd* zoospores (Fig. 5), which matches the timing of fungal germ tube development during invasion [17]. Assessing the timing and extent of damage via DAPI staining may therefore be useful for quantifying virulence of different *Bd* isolates, as these can differ in their ability to invade A6 cells and exhibit endobiotic (intracellular) growth [30]. DAPI will also stain non-viable *Bd* zoospores, but this issue can be resolved by capturing images at a low magnification (eg: 40x) as the zoospore nuclei are much smaller than A6 cells, as shown by the lack of signal in the 27 °C treatment (Fig. 4a). Other methods to quantify cell health in this model are complicated because they measure both the host cell and the pathogen. As most host cellular functions are expected to decrease as pathogen burden and function increases, this could obscure the effect. For example, crystal violet stains A6 cells, and can be used to estimate cell surface area or volume. However, quantification of cellular staining is confounded as it also strongly stains *Bd* zoosporangia. As *Bd* zoosporangia are much smaller than A6 cells, we were still able to detect a reduction in A6 coverage using image analysis, however this could be refined via the use of image processing software to subtract the stained *Bd*. Measurement of A6 cell health using MTT required including anti-fungal treatment of elevated temperatures coupled with an antifungal drug to reduce interference from *Bd* metabolic activity before measuring cellular viability. This highlights the importance of considering both the host and pathogen separately when evaluating new methods for infection models.

Interestingly, we noted that exposure to a high concentration of *Bd* zoospores caused a slight but significant reduction in A6 cell area, even at temperatures that inhibit *Bd* growth (Fig. 7). Both *Bd* and *Bsal* can produce metabolites that negatively affect cells [31,32]. For example, survival and proliferation of amphibian lymphocytes is impacted by *Bd* secretions, however this effect is limited to zoosporangia and not pure zoospores [33]. Our results suggest that *Bd* zoospores may produce metabolites that negatively affect A6 cell growth if densities are high enough.

In this study, we have demonstrated a range of possible methods to quantify infection and disease utilising chytridiomycosis cell infection models. However, not all these methods will be suitable, or may require optimisation depending on specific experimental design. For example, MTT requires a careful balance of deactivating *Bd* zoosporangia without harming the A6 cells, which may be difficult if A6 cells are under additional stress. Our DAPI staining protocol may not be suitable for experiments where fully grown zoosporangia are killed, as they will also be stained (Supplementary Fig. 2).

Endobiotic growth within A6 cells has been found to be associated with *Bd* virulence [30]. The *Bd* isolates used in these experiments belong to the virulent Global Pandemic Lineage (GPL) and displayed both epibiotic and endobiotic growth. Due to the strong fluorescent signal of tdTomato, the transparency of A6 cells, and the sensitivity of fluorescent microscopes, flow cytometers and microplate readers, we assume that endobiotic growth was captured by these methods. The future development of methods to quantify epibiotic and endobiotic growth would further strengthen this toolbox of methods and allow more in-depth comparison of *Bd* virulence.

Our findings establish a robust platform to assess and quantify *Bd* infection in amphibian host cells. This capability will not only enhance our understanding of host-pathogen-environment interactions but will also advance the evaluation and development of novel mitigation strategies against chytridiomycosis. Cell infection models could be employed to investigate 1) fungal virulence mechanisms or host resistance factors through targeted gene manipulations [34,35], 2) the interactions between *Bd* and other amphibian pathogens [36], or 3) host/pathogen gene expression.

Although *in vitro* models utilizing the *X. laevis* A6 kidney epithelial cells can simulate key features of natural infections observed in amphibian skin [17,18], amphibian epidermal cell lines would provide a more realistic model. Recent progress with generating frog skin epithelial cell lines [37] opens opportunities to enhance the biological relevance and applicability of the model. Moreover, the development of epidermal cell lines from various susceptible and resistant amphibian species could permit comparative studies on cellular defences. Furthermore, *in vitro* systems, such as transwell systems and complex organ-on-chips methods are widely used in human fungal disease research to mimic complex host tissue environments [1]. Subsequent to the development of an amphibian epidermal cell line, generating a stratified, keratinising skin organoid would provide an ideal platform for studying host-pathogen interactions and testing therapeutic interventions for chytridiomycosis.

## Supplementary Material

1

## Figures and Tables

**Table 1:** Summary of methods useful for quantification of chytridiomycosis parameters in an *in vitro* cell infection model. Methods are ranked on the following: Are the basic materials inexpensive? Can the data be obtained quickly? Can the protocol be repeated on the same samples over time? Does the method avoid the opportunity for bias? Can the data be obtained using standard laboratory equipment? Can the method detect subtle differences?

Parameter and Method	Cheap	Quick	Non-destructive	Reduces Bias	Basic lab equipment	Sensitive
*Bd* load - Image J	✓	X	✓	X	X	✓
*Bd* load - Spectrophotometry	✓	✓	✓	✓	X	X
Infection - Flow cytometry	✓	X	X	✓	X	✓
Cell damage - DAPI	✓	✓	X	X	X	✓
Cell size - Crystal Violet	✓	✓	X	X	✓	X
Cell activity - MTT	X	X	X	✓	✓	✓

## Data Availability

Data will be made available on request.

## Appendix A. Supplementary material

Supplementary data to this article can be found online at https://doi.org/10.1016/j.ymeth.2026.02.006.
