# Supplementary material for: Using fluorescent in vitro amphibian cell infection models to quantify pathogenicity of Batrachochytrium dendrobatidis
Source: Methods. Author manuscript; Available in PMC 2026 Jun 3. (PMC13231286; doi:10.1016/j.ymeth.2026.02.006)
Supplement: 1 [file NIHMS2177851-supplement-1.docx]

Using fluorescent *in vitro* amphibian cell infection models to quantify *Batrachochytrium dendrobatidis* pathogenicity

Kashmini K Sumanasekera, Lee Berger, Andrea L Vu, Jacques Robert, Nazia Akram, Lee F Skerratt, Francisco De Jesús Andino, Rebecca Webb

Supplemental data


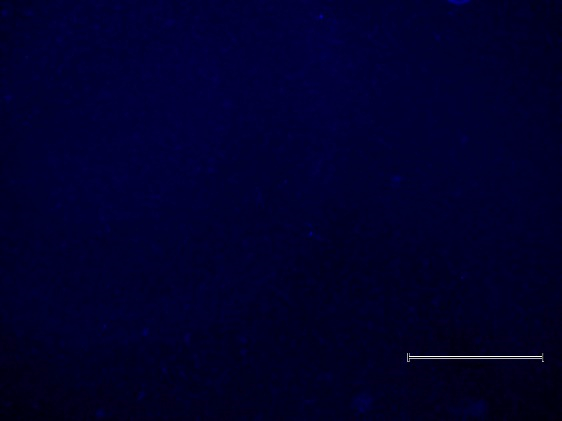

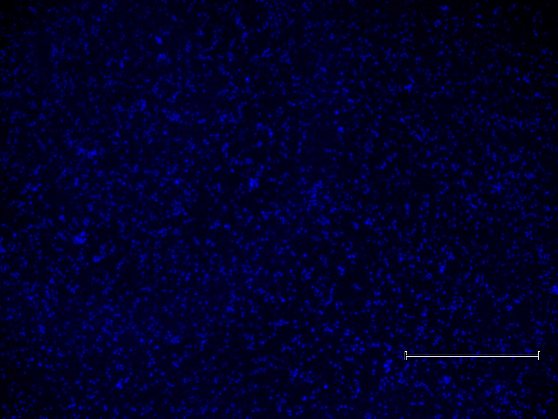


B

A

**Figure S1**-DAPI staining of live and dead *Bd* zoosporangia. Zoospores (50,000 per well) were added to in 96 well plates in 100µL TGhL and grown at 19°C for 3 days. Zoosporangia were stained with 0.16 µg/mL DAPI for 5 mins with or without prior fixation with formalin. One representative image of live (A) or dead (B) zoosporangia after DAPI staining, scale bar is 750 µm.


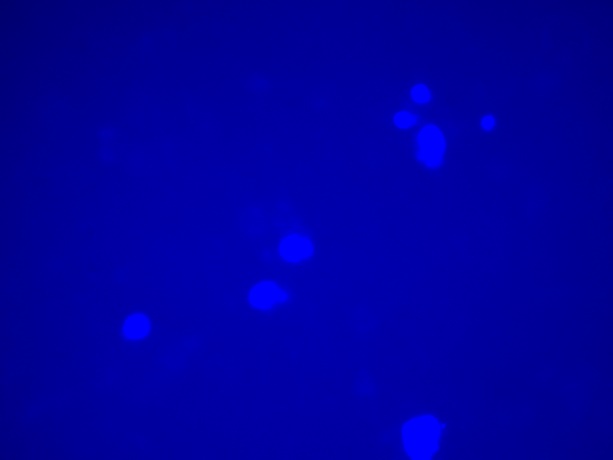

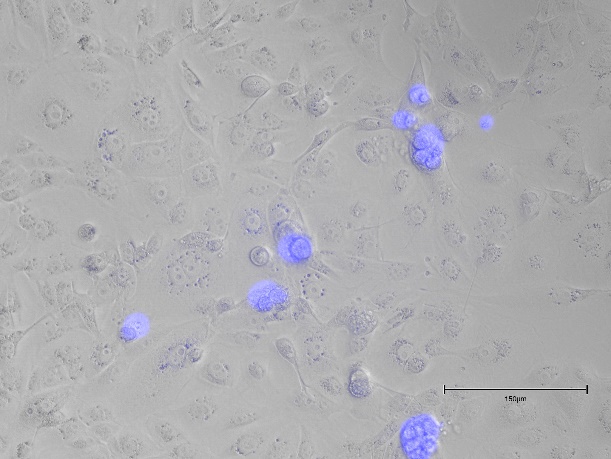


B

A

**Figure S2**-DAPI staining of dead *Bd* zoosporangia within A6 cells. *X. laevis* A6 cells were infected with *Bd* zoospores at 1 MOI and incubated 19°C for 3 d, then treated with 5 µg/ml Terbinafine hydrochloride (Lamisil) for 18 h. The infected cells were stained with 0.16 µg/mL DAPI for 5 mins. One representative image of the DAPI channel (A) and merged DAPI and TRANS channels (B) scale bar is 150 µm.

**Figure S3-** Effectiveness of antifungal treatment on A6 MTT assay.

*X. laevis* A6 cells were infected with *Bd* zoospores at 0 or 4 MOI and incubated 19°C for 7 d. Infected wells were either treated with 5 µg/ml Terbinafine hydrochloride and incubated at 30 °C for 18 hr to reduce interference from live *Bd*,or left at 19°C without antifungal treatment. A6 cell metabolic activity was estimated using a commercial MTT assay (CyQUANT™). The relative absorbance of three technical replicates is shown with standard deviations.
